# Kinetic Analysis of High-Temperature Sunflower Oil Peroxidation Inhibited by the Major Families of Phenolic Antioxidants Unveils the Extraordinary Activity of 1,4-Hydroquinones

**DOI:** 10.3390/antiox11112142

**Published:** 2022-10-29

**Authors:** Fabio Mollica, Lucia Bonoldi, Riccardo Amorati

**Affiliations:** 1 Department of Chemistry “G. Ciamician”, University of Bologna, Via S. Giacomo 11, I-40126 Bologna, Italy; 2 Research and Technological Innovation Department, Eni SpA, Via F. Maritano 26, I-20097 San Donato Milanese, Italy

**Keywords:** peroxidation, kinetic, bond dissociation, tocopherol, polyphenols, EPR, sunflower oil, peroxyl radical, oxygen consumption, phenoxyl radical

## Abstract

Peroxidation of vegetable oils represents a major problem for the food and biodiesel industries, and it is greatly accelerated by oil degree of unsaturation and by temperature increase. Phenols represent the most common additives used to counteract oil peroxidation, however clear structure-activity relationships at high temperatures are not available. We report, herein, a kinetic study of O_2_ consumption during spontaneous peroxidation of sunflower oil at 130 °C in the presence of 18 antioxidants belonging to the main families of natural and synthetic phenols, including α-tocopherol, alkylphenols (BHT, BHA), hydroquinones (TBHD), catechols (quercetin, catechin) and gallates. Results show that TBHQ provide the best protection in terms of induction period (*IP*) duration and O_2_ consumption rate. EPR spectroscopy demonstrated that the inhibition activity is negatively correlated to the stability of the phenoxyl radical of the antioxidant (A^•^), suggesting that chain propagation with linoleate (RH) moieties A^•^ + RH → AH + R^•^ decreases the efficacy of those antioxidants forming persistent A^•^ radicals. These results provide important information to optimize the antioxidant activity of phenols and of novel phenol-based materials.

## 1. Introduction

Lipid peroxidation is a main problem for edible oil utilization, and for the long-term preservation of foods rich in polyunsaturated lipids. The formation of hydroperoxides and other oxygenated products like epoxides, aldehydes, ketones, diols, etc. are correlated with off-flavor, insoluble deposits, and with the formation of potentially toxic compounds [1]. Besides, recycling used oil as feedstock for biorefineries requires the control of oxidation and polymerization processes that can cause corrosion and clogging problems [2]. It is therefore understandable why the development of novel and more effective ways to counteract lipid peroxidation is still an active research field. Phenols represent the most important class of natural and artificial antioxidants, and therefore it is not surprising that many recent studies are based on the manipulation and optimization of their characteristics. For example, in the emerging field of nano-antioxidants [3,4], natural phenolic polymers like lignin [5] and melanin-like materials [6,7] are among the most promising radical-trapping platforms. Besides, many natural-based waste products currently investigated as a source of antioxidants are particularly rich in phenols, like for instance, cashew-nut shell liquid [8], or olive mill wastewater [9].

Knowledge of the factors that determine the efficacy of phenolic antioxidants is of fundamental importance to guide the research toward significant advancements. While several experimental and theoretical studies performed in the past decades have clarified the chemico-physical basis of the antioxidant activity of phenols [10,11], the prediction of the efficacy in real systems (such as in emulsions, etc.) remains challenging [12]. The behavior of phenols as antioxidants for oils at high temperatures is particularly important because these conditions are found not only in many technological applications such as cooking and oil processing but also in accelerated stability tests such as Rancimat [13]. Previous studies reported the effect of phenols as stabilizers toward oil (or fatty acids methyl esters, FAME) peroxidation at high temperatures, but rarely they are performed by comparing a significant number of different phenols and, to the best of our knowledge, never report a chemical explanation of the results. For example, Dodos et al. compared the activity of ten phenolic antioxidants in the stability at 110 °C of FAME from soybean oil (rich in linoleic acid) by using Rancimat equipment and found the longest induction period (*IP*) in the case of *tert*-butyl hydroquinone (TBHQ), while monophenols like BHT and BHA did not show significant effects [14]. R. Becker and A. Knorr, in a study of the oxidation of rapeseed oil at 130 °C, showed that, among several investigated phenols, 2,5-di-*tert*-butyl-hydroquinone had the best activity in terms of *IP* [15]. However, the reason for this activity ranking remained unclear.

We, therefore, decided to study the antioxidant activity of 18 phenols, chosen among the main families of natural and synthetic phenols, with the aim of deriving a structure/activity relationship and understanding the mechanism of inhibition of lipid peroxidation. We used a recently developed method consisting of real-time measuring of the O_2_ consumption in the headspace of a purified oil sample, vigorously stirred at 130 °C [16,17]. This approach minimizes the loss of the antioxidant by evaporation and allows determining not only the *IP* but also the slope of the O_2_ consumption during the *IP* (−*d*[*O*_2_]_inh_/*dt* = *R*_inh_). While IP is a measure of the lifetime of the antioxidants in the selected environment, *R*_inh_ correlates with the rate of reaction of the antioxidant with free radicals, i.e., its efficacy in slowing down oxidation [16]. The collected results are analyzed by considering the accepted mechanisms underlying lipid peroxidation. With the help of electron paramagnetic resonance (EPR) spectroscopy, we were able to detect, in specific cases, the presence of stable radicals, affording a plausible explanation of the efficacy order. Our results, besides confirming the previously unexplained high activity of hydroquinones, provide a predictive rationalization based on the behavior of the radicals derived from phenols under peroxidation conditions.

## 2. Materials and Methods

### 2.1. Materials

All chemicals and solvents were commercially available (Merk, Sigma-Aldrich, Milan, Italy). *Para*-methoxyphenol (**1**), 2-*tert*-butyl-4-methoxyphenol (**2**), 2,6-di-*tert*-butyl-4-methylphenol (BHT) (**3**), 2,4,6-trimethylphenol (**4**), 2-methoxy-4-ethylphenol (**5**), 2,6-dimethoxy-4-allylphenol (**6**), (±)-α-Tocopherol (**7**), 2,2,5,7,8-pentamethyl-6-chromanol (**8**), *para*-hydroquinone (**9**), *tert*-butylhydroquinone (**10**), 2,5-di-*tert*-butylhydroquinone (**11**), *para*-benzoquinone (**12**), *tert*-butylbenzoquinone (**13**), 2,5-di-*tert*-butylbenzoquinone (**14**), catechol (**15**), 4-methylcatechol (**16**), 4-*tert*-butylcatechol (**17**), caffeic acid (**18**), catechin (**19**), quercetin (**20**), propyl gallate (**21**), triacetin were of the highest available purity and were used as received. Antioxidant concentrations are % *w*/*w*. Solvents were HPLC-grade and were used without further purification. High-linoleic sunflower oil was purchased from a local market. Stripped sunflower oil (SSO) was prepared as previously reported by percolation on basic alumina and by treatment with activated carbon [17]. The SSO composition was determined by ^1^H-NMR [18]: polyunsaturated chains 59.4 mol %, monounsaturated chains 27.3 mol %, saturated chains 13.3 mol %.

### 2.2. Autoxidation Experiments

Oxygen consumption during the sunflower oil autoxidation was measured by an O_2_-sensitive optical probe (Pyroscience GmbH, Aachen, Germany) in a sealed apparatus previously described [16]. Briefly, 2.00 g of oil containing variable amounts of the antioxidants is introduced into a 10 mL round-bottomed flask with a magnetic stir bar connected to a miniaturized water-cooled condenser to keep the temperature of the O_2_-sensitive probe below 40 °C (total internal volume 29 mL). The sample is kept under vigorous stirring in a silicone bath at 130 °C. The O_2_ probe is calibrated in air before each experiment. The amount of consumed O_2_ is calculated from the internal volume of the apparatus. The experiments with peroxidized SSO were performed by oxidizing SSO at 130 °C measuring O_2_ uptake. Then the sample was aerated, and a small volume of a concentrated octanol solution of **10** was added.

### 2.3. Analysis of Antioxidant Stability

The experiments were performed under the same conditions as the autoxidation experiments, except for the use of triacetin, which is resistant to oxidation at 130 °C, in place of SSO. The concentrations of the antioxidants were determined by HPLC separation coupled to a photodiode array (DAD) UV-vis detection and the flow was set to 0.9 mL/min. The column was a Sepachrom C_18_, 5 μm, 250 mm × 4.1 mm. The mobile phase was an isocratic mixture of MeCN/H_2_O 60:40 for **9**, **10** and **11**; MeCN/H_2_O 90:10 for **2**; MeCN/H_2_O + 3% formic acid 50:50 for **21**; MeCN/MeOH 90:10 for **8**.

### 2.4. EPR Analysis

The determination of the concentration of the radicals of **2**, **3**, **7,** and **10** was performed by an X-band electron paramagnetic resonance (EPR) spectrometer (Elexsys 500, Bruker, Billerica, MA, USA) equipped with a variable temperature dewar placed in the resonant cavity. Suprasil quartz EPR tubes were filled with the pure oil samples (no dilution) up to 4 cm and thermostatted at 40 °C by nitrogen gas flowing in the dewar. The small quantity of oil ensured a homogeneous temperature inside the analyzed sample. Typical instrumental settings were: microwave power = 10 mW, amplitude = 2 G, 64 accumulations. The radical concentration was obtained from the double integration of the EPR signal multiplied by the inverse temperature in Kelvin degrees to correct for the Curie temperature dependence and calibrated with the signal of the strong-pitch standard from Bruker. The spectra were simulated with the software Winsim [19], freely available from the Public Electron paramagnetic resonance Software Tools (PEST) from NIEHS (National Institue of Environmental Health Sciences). The radicals were identified as the phenoxyl radicals of the investigated antioxidants from the agreement of their spectral parameters (hyperfine coupling constants, *a*, and *g*-factors) with those reported in the literature [20,21] or with those expected from their structure [22].

### 2.5. Statistical Analysis

O_2_-consumption experiments and HPLC analysis were performed in duplicate. The values are expressed as the average ± SD (standard deviation). Student’s *t*-test was applied for comparisons between two mean values. Differences were considered to be of significance when *p* was ≤ 0.05.

## 3. Results

### 3.1. Autoxidation Experiments

**Figure 1:**
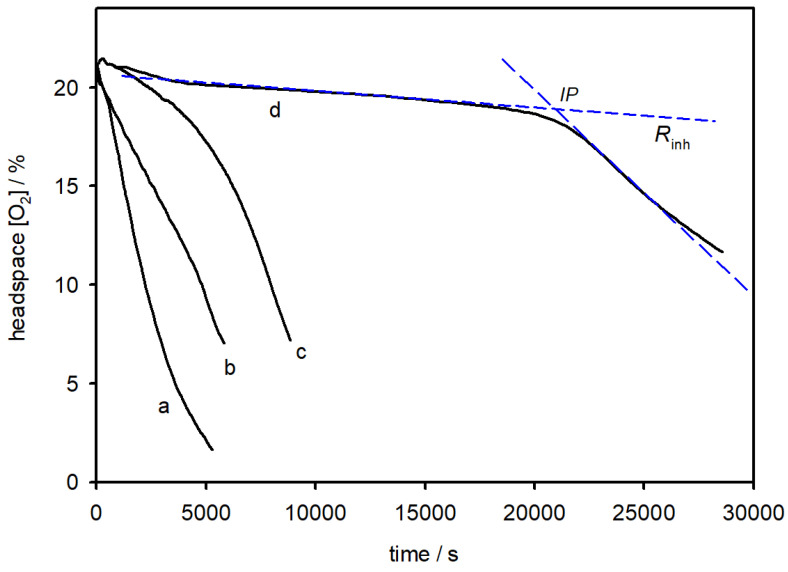
O_2_ consumption during the autoxidation of SSO at 130 °C without antioxidants (**a**) or in the presence of 0.1% (*w*/*w*) of α-tocopherol (**b**), BHT (**c**) and TBHQ (**d**).

Stripped high-linoleic sunflower oil (SSO), in the absence of antioxidants, showed an initial linear O_2_ consumption, followed by a decrease in the oxidation rate with the decline of O_2_ concentration in the headspace (Figure 1, trace a).

**Scheme 1:**
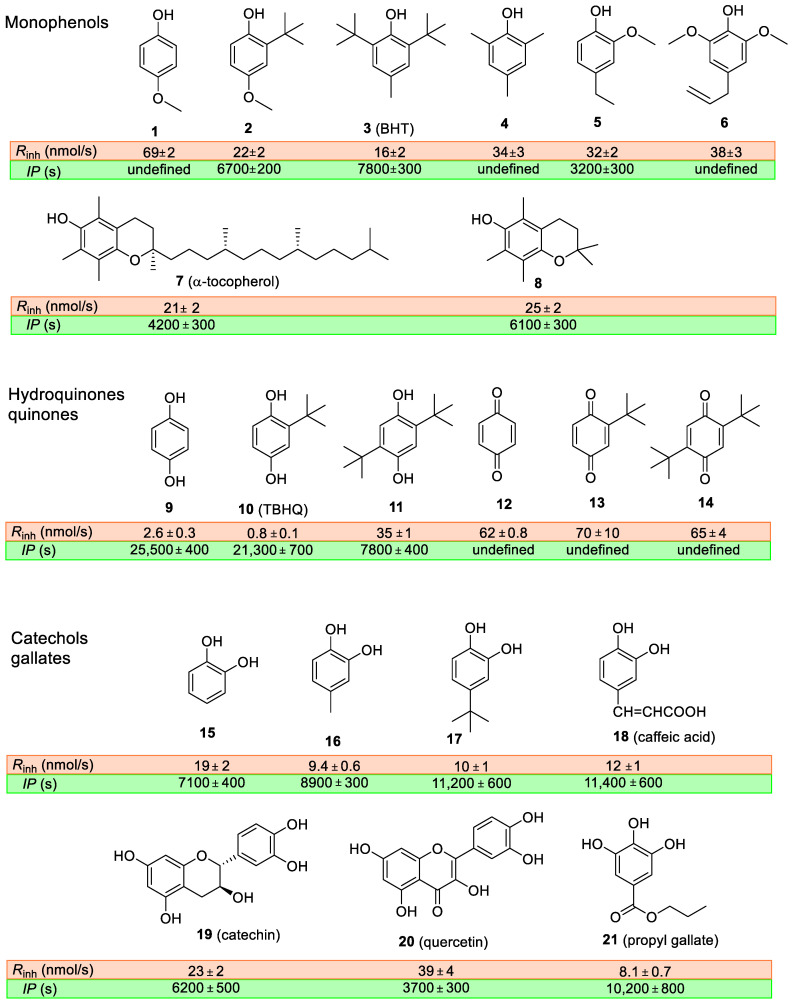
Antioxidant effect of the mono-phenols: oxygen consumption rate in the inhibited period (*R*_inh_) and duration of the inhibited period *IP*. The slope without antioxidants was 70 ± 8 nmol/s. Undefined *IP* indicates that no induction period is present.

In the presence of the antioxidants, the autoxidation onset of SSO was retarded for a variable period of time (induction period, *IP*), after which the O_2_ consumption was faster. The minimum slope of O_2_ consumption during the inhibited or retarded period (inhibited rate, *R*_inh_) and the duration of the inhibition *IP* were taken as indicators of the efficacy of the antioxidants. In Figure 1, some typical O_2_ consumption curves and the derivation of *R*_inh_ and *IP* are reported. By following this procedure, we analyzed several natural and synthetic phenolic antioxidants all at the identical concentration of 0.1% *w*/*w*, and the results are reported in Scheme 1. The antioxidants were divided into three families depending on the number of OH groups and their relative position because, as will be evident in the discussion of the results, this feature influences the fate of the radical of the antioxidant and the overall antioxidant efficacy.

Monophenols, characterized by the presence of only one phenolic OH group, include α-tocopherol (**7**) and its synthetic analog **8**, two *ortho*-methoxy phenols (**5** and **6**) having a structural relationship with natural compounds like sinapic acid and eugenol, the well-known antioxidant additives BHT (**3**) and BHA (**2**), and other alkylated or methoxylated phenols used for comparison purposes (**1** and **4**).

Hydroquinones are characterized by the presence of two OH groups in *para* position, and a variable number of alkyl substituents. They include unsubstituted hydroquinone (**9**), *tert*-butyl hydroquinone (TBHQ, **10**), and 2,5-di-*tert*-butyl hydroquinone (**11**). The corresponding oxidized *para*-quinones (**12**–**14**) were also investigated to ascertain the influence of these compounds on the radical chain, as it was reported that they may act as alkyl (R^•^) [23] or HOO^•^ radical traps [24].

The last group is represented by catechols, having at least two OH groups in *ortho* position. They include the natural antioxidants caffeic acid (**18**), catechin (**19**), and quercetin (**20**) as well as unsubstituted catechol (**15**) and the alkylated catechols **16** and **17**, while gallates are represented by the antioxidant additive propyl gallate (**21**).

**Figure 2:**
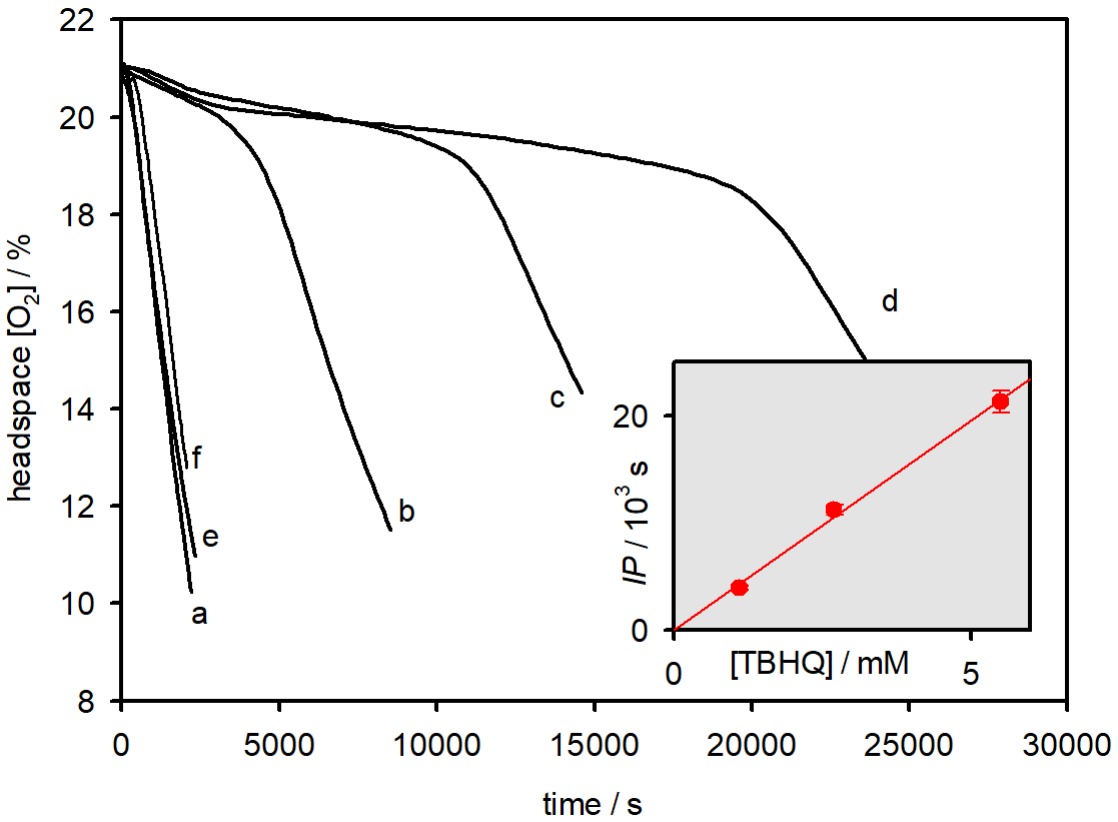
O_2_ consumption during the autoxidation of SSO at 130 °C without antioxidants (**a**) or in the presence of **10**: 0.02% (**b**), 0.05% (**c**) and 0.1% (**d**), or **13**, 0.1% (**e**). O_2_ consumption of peroxidized SSO in the presence of **10**, 0.02% (**f**). The inset reports the relationship between the **10** concentrations and *IP*. Some error bars are smaller than the symbol size.

With the exception of 4-methoxyphenol (**1**) and of benzoquinones **12**–**14**, all the investigated compounds had some retarding effect on SSO peroxidation. However, the only antioxidants that were able to afford a strong inhibition for a long time were the hydroquinones **9** and **10**. In fact, they were able to reduce 30 and 100 times the slope of O_2_ consumption, respectively, while the *IP* was about 2.5 × 10^4^ s, that is, considerably longer than that of the best non-hydroquinone antioxidants like propyl gallate. If considered on a molar basis, **10** displayed the highest radical trapping stoichiometry, that is the number of radicals trapped by each antioxidant molecule, among all investigated phenols. Because of its exceptional characteristics, the *IP* dependence on the concentration of **10** was investigated in detail, as shown in Figure 2. The inset in this figure shows that in the concentration range considered, *IP* is linearly dependent on **10** concentrations.

In order to test the effect of hydroperoxides on the *IP* of **10**, we studied the effect of **10** (0.02%) in an SSO sample that was previously oxidized without any antioxidant, until reaching a [ROOH] ≈ 200 mM, assuming that all consumed O_2_ is transformed into ROOH. The results show that the antioxidant effect of **10** almost disappears (compare plot f with b, Figure 2), indicating a major effect of peroxide accumulation on *IP*.

### 3.2. EPR Experiments

**Figure 3:**
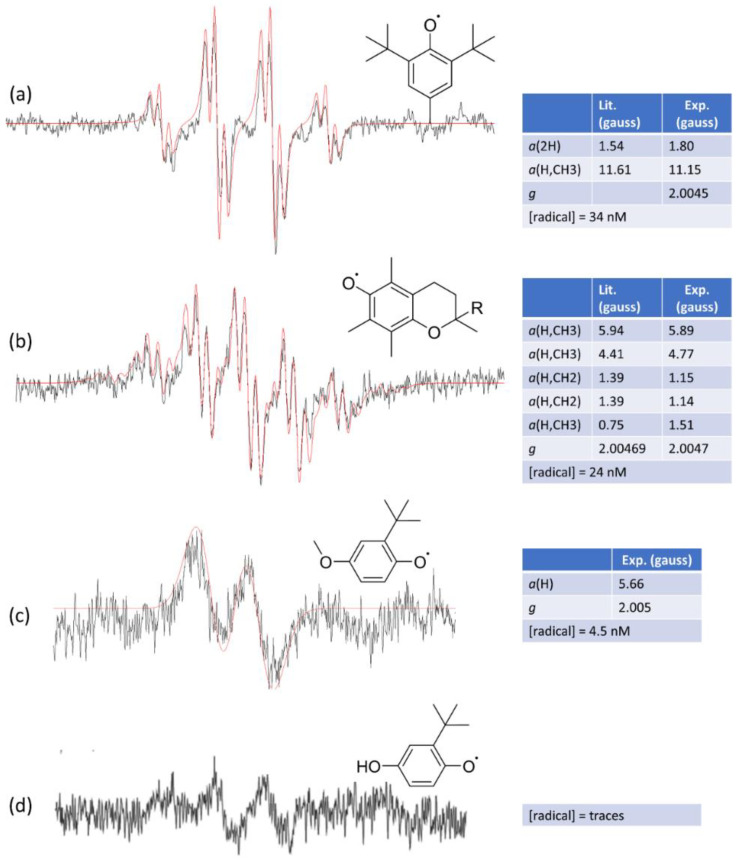
Experimental and simulated (red lines) EPR spectra of SSO after the addition of 0.1% at 40 °C of **3** (**a**), **7** (**b**), **2** (**c**), **10** (**d**). The coupling constants (*a*), the *g*-factor, and the radical concentrations (estimated error ± 50%) are also reported.

To investigate the relationship between the stability of the radicals and the antioxidant activities of the investigated phenols, we measured the electron paramagnetic resonance (EPR) spectra of **2**, **3**, **4,** and **10** at 0.1% in SSO under air. Aerated oil samples containing the antioxidants were introduced in the EPR cavity and heated at 40 °C. This temperature was chosen as the best compromise to optimize the signal-to-noise ratio. In the case of α-tocopherol (**7**) and BHT (**3**), the signal arising from their phenoxyl radicals could be clearly detected (see Figure 3a,b), while in the case of **2** the signal was weak, and in the case of **10** only traces of the radical could be evidenced. The identity of the radicals was ascertained by analyzing the experimental spectra by numerical simulation as shown by the red lines in Figure 3. This procedure afforded coupling constants (*a*) and *g* factors that were in good agreement with those reported in the literature for the respective phenoxyl radicals [19,20,21,22]. The radical concentrations (see Figure 3) were in the nanomolar range and, considering the experimental error, the radical stability decreased in the order: **3** ≈ **7** > **2** >> **10**.

### 3.3. Stability of Antioxidants

**Figure 4:**
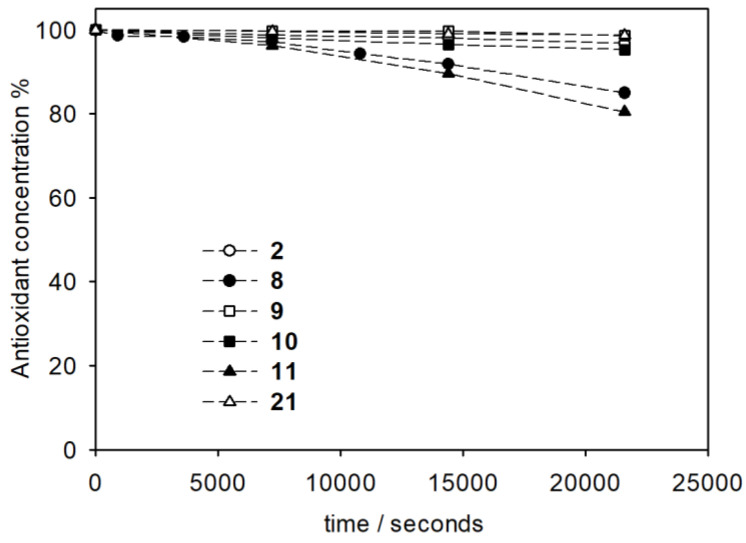
Stability at 130 °C of selected phenols in triacetin under air. Error bars are smaller than the symbol size.

The stability at 130 °C was investigated to understand the role of phenol degradation on the duration of the inhibited period. Selected antioxidants (see Figure 4) were dissolved in triacetin, an oxidation-resistant triacylglycerol having no unsaturations, and were placed in the same reactor used in the peroxidation experiments. Then, their concentration was measured by HPLC-DAD analysis at time intervals for 6 h. The hydroquinones **9–11** were considered in order to clarify the excellent antioxidant behavior of **9** and **10** in SSO autoxidation experiments, as opposed to the relatively small efficacy of the dialkylated analog **11**. The monophenol **2** and propyl gallate (**21**) were included for comparison purposes, while **8** was selected to investigate the behavior of α-tocopherol.

We found that in all cases the concentration remained approximately constant, except for the dialkylated hydroquinone **11** and the α-tocopherol analog **8**, whose concentrations, however, decreased only by 20% and 15%, respectively, after 6 h (21.6 × 10^3^ s). If considering that in the SSO autoxidation experiments their *IP* was 7.7 × 10^3^ s and 6.6 × 10^3^ s, respectively, even in the case of the least stable phenols **8** and **11**, thermal degradation plays a negligible role in the duration of the inhibition. In addition, these experiments also demonstrated that the antioxidant loss by evaporation was negligible even in the case of the low molecular weight phenols **2** and **8**.

## 4. Discussion

**Scheme 2:**
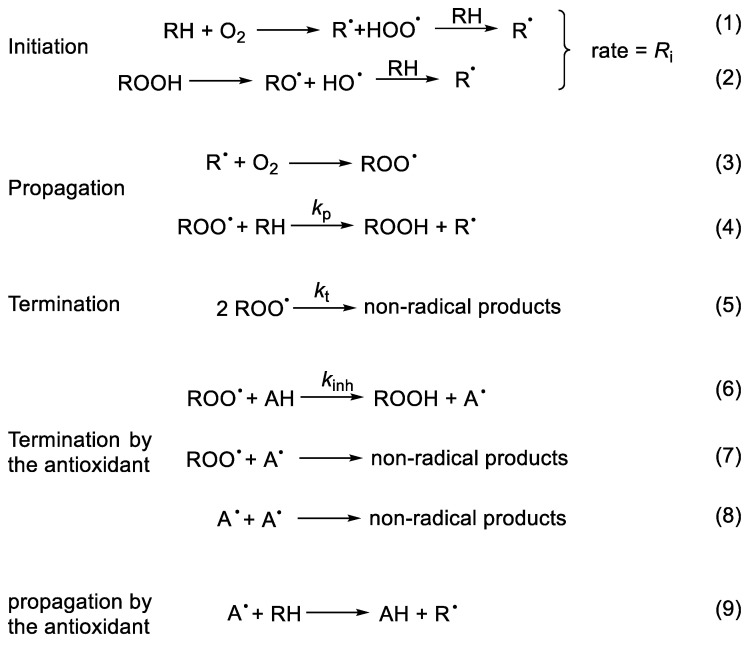
Reactions occurring during SSO autoxidation in the presence of antioxidants. RH = polyunsaturated triacylglycerols, AH = antioxidant.

The results collected herein show, in a comprehensive manner, the effect of the major families of phenolic antioxidants on the oxidation of high-linoleic sunflower oil at 130 °C. Despite their structural similarity, phenolic antioxidants display highly variable inhibition capability, as expressed by the reduction of the *R*_inh_ rate, and by the duration of the inhibited period *IP*. Hydroquinones **9** and **10** display the highest inhibition, while other phenols having a very similar structure, such as their methylated analogs **1** and **2**, are much less active. To understand these results, reactions 1–9 occurring during SSO peroxidation have to be considered (Scheme 2) [10,25].

The initiation step (reactions 1 and 2) consists of the formation of radicals in the system with an overall rate *R*_i_. Reactions 3–5 describe the processes occurring in not-inhibited autoxidation, where hydrogen-atom abstraction (reaction 4) occurs (mainly) at the bis-allylic position of the linoleate residues and the radical chain propagates with rate constant *k*_p_ [10].

The initiation process may occur either through the direct reaction of activated C-H (usually bis-allylic ones) with O_2_ (reaction 1) or by decomposition of hydroperoxides ROOH (reaction 2) [25,26]. Considering that only a small fraction of bis-allylic groups is consumed, the first mechanism is expected to decline only with O_2_ concentration, while the second mechanism is marginal at the beginning of the reaction, or in the presence of strong antioxidants, but becomes more and more important as the autoxidation proceeds and hydroperoxides are accumulated.

The *R*_i_ parameter has a great influence on *IP* because the faster radicals are produced in the system, the faster the antioxidants are consumed, and the *IP* is shortened. By applying the steady state approximation, which states that in radical chains the concentration of radical species is constant and that the overall rate of the formation of radicals must be equal to that of their disappearance, and considering *R*_i_ is constant during the inhibited period, the following relation applies: *IP* = *n*[AH]/*R*_i_, where [AH] is the initial concentration of the antioxidant and *n* is the number of radicals trapped by each AH. At low temperatures, phenols quench ROO^•^ by reactions 6–8 and are usually characterized by *n* ≈ 2 [10,25].

In the case of **10,** which is one of the best antioxidants analyzed here, the *IP* increases linearly with **10** concentrations (Figure 2) suggesting that *R*_i_ is constant throughout the duration of the inhibited period of all three experiments shown in Figure 2, otherwise reduction in the *IP* should have been observed caused by the increase of hydroperoxides. On the other hand, when **10** is used as an inhibitor of an SSO sample already containing a high level of hydroperoxides, no antioxidant effect is observed, suggesting a major role of ROOH in increasing *R*_i_.

In the case where *R*_i_ would be only due to the bulk oil’s original composition (i.e., mainly the bis-allilic hydrogen concentration) in all samples, *IP* would be approximately the same throughout the investigated phenols. However, it turned out not to be so: *IP* varied significantly depending on the antioxidant (see values in Scheme 1): antioxidants causing a small reduction of O_2_ consumption rate (i.e., those having high *R*_inh_) were also characterized by low *IP*. To explain this effect, we hypothesized that in the case of weak antioxidants also, hydroperoxides may contribute to *R*_i_, increasing its value with respect to the pristine bulk phase. The O_2_ consumed during the inhibited period of weak antioxidants is converted into hydroperoxides, that in turn increase *R*_i_, causing a shortening of *IP*.

Reactions 6–8 account for the action of phenolic radical-trapping antioxidants, consisting in H-atom donation to the chain-carrying peroxyl radicals ROO^•^, with a kinetic rate constant *k*_inh_, followed by the decay of A^•^ with another radical (reactions 7 and 8) [10,25]. The slope of the inhibited period, *R*_inh_, is inversely proportional to *k*_inh_. In the steady state approximation, *R*_in_ = −*d*[*O*_2_]/*dt* = −(*k_p_*[RH]*R_i_*)/(*n* *k*_inh_[AH]) [10].

In addition, it must also be considered that, if the decay of the A^•^ radical is not efficient, it can react with the substrate, propagating the oxidative chain via reaction 9. This reaction, although regenerating an active antioxidant molecule AH, has, however, the undesired effect of opposing reaction 6, thus contributing to the formation of hydroperoxides and increasing *R*_inh_.

Overall, this reaction scheme shows that good antioxidants must have a high *k*_inh_ value (reaction 6) and must decay efficiently to avoid the onset of reaction 9.

**Scheme 3:**
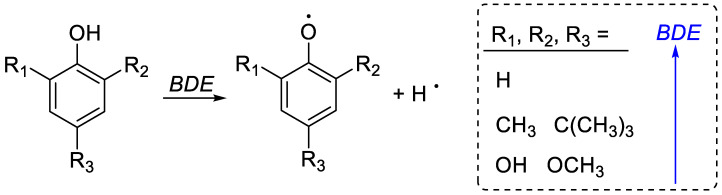
Substituent effect on the O-H bond dissociation enthalpy (*BDE*) of phenols.

It is well known that, in order to have high *k*_inh_ values, phenols must possess electron-donating (ED) substituents in *ortho* and *para* positions with respect to the reactive OH group (see Scheme 3) [27]. ED substituents decrease the bond dissociation enthalpy (*BDE*) of the OH group and thus more easily render the H-atom abstraction by ROO^•^ radicals. The substituent effect is proportional to the ED power of the substituents (OMe = OH > Me = *^t^*Bu > H) and is modulated by the bulkiness of the substituents and by the presence of intramolecular H-bonds [27]. For example, *ortho*-methoxy phenols have a low reactivity toward ROO^•^ because the OH group is engaged in a strong H-bond with the OMe substituent [28]. Accordingly, phenols having only one ED substituent (like **1**) or *ortho*-methoxy substituents (like **5** and **6**), were poor inhibitors of SSO autoxidation.

However, these rules seem not to apply in every case. For instance, **2** and **10** which have the same number of substituents with the same ED ability, show instead very different antioxidant effects, both in terms of *R*_inh_ and of *IP*. In addition, α-tocopherol (**7**) and its analog **8** which have the lowest *BDE*(OH) among the investigated phenol [27] are not the most active ones.

**Scheme 4:**
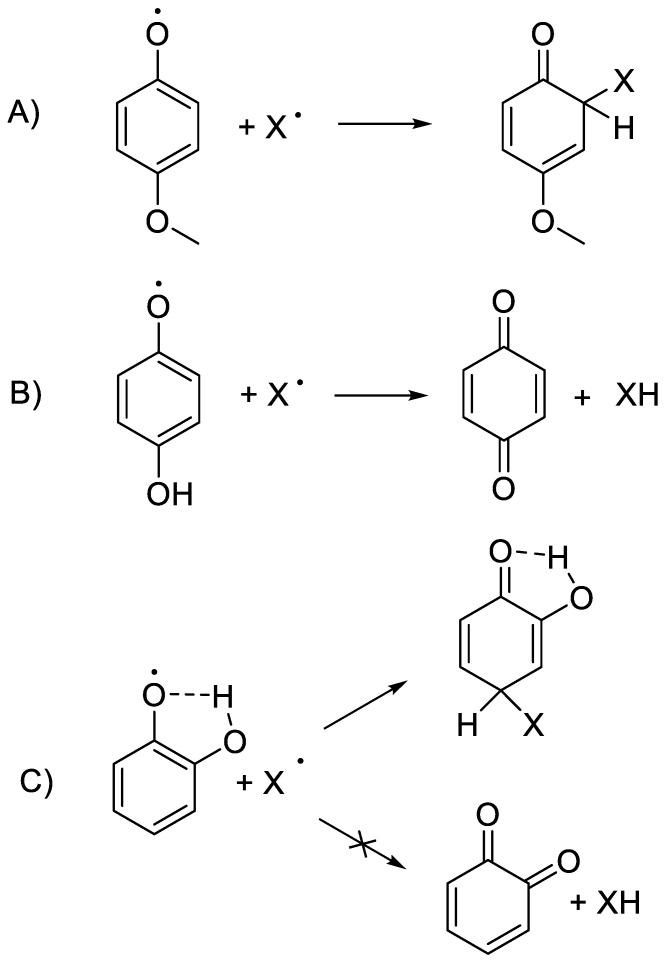
Decay pathways of the phenoxyl radicals formed by monophenols (**A**), *para*-hydroquinones (**B**), and catechols (**C**) upon reaction with a generic radical X^•^.

As stability experiments showed that the spontaneous decay of the antioxidants at 130 °C is negligible (see Figure 4), these differences must be explained by considering the interplay between reactions 6–9. EPR experiments were performed to investigate this stability. They showed that the stability of the radicals of the antioxidants increases with the number and bulkiness of the substituents in *ortho* and *para* positions to the phenolic OH in the order **3** ≈ **7** > **2**, while the stability of the radical from **10** is extremely low. This result can be explained by the tendency of phenoxyl radicals to decay mainly by radical-radical addition by using the *ortho* and *para* positions, as shown in Scheme 4A [22]. If these positions contain alkyl or methoxy substituents, the decay is slow and possibly reversible.

On the other hand, the 4-hydroxylphenoxyl radical formed by **10** (see Scheme 4B) is very unstable because it decays by H-atom transfer with another radical forming the *para*-benzoquinone **13** and is therefore unable to propagate the oxidation via reaction 9. Interestingly, **13** is the main product that is formed in oxidized soybean oil stabilized by **10** [29].

The instability ranking experimentally observed by EPR follows the antioxidant ranking, proving that persistent radicals can behave detrimentally, because of their contribution to the chain propagation mechanism.

In this context, the disappointing behavior of catechol and gallates was unexpected, because also in their case, the phenoxyl radical may potentially decay by forming an *ortho*-quinone. However, the present results rather suggest that the OH groups in the phenoxyl radicals from catechols and gallates are not easily transferred to another radical, likely because they are involved in a strong intramolecular H-bond with the -O^•^ group (see Scheme 4C) [30].

**Figure 5:**
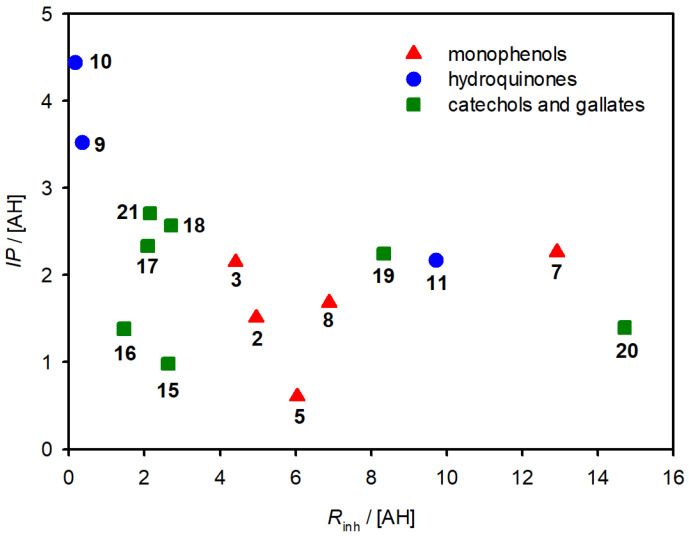
Relation between *R*_inh_ and *IP* divided by the molar antioxidant concentration (in arbitrary units). Antioxidants with undefined *IP* were omitted.

In agreement with this view of reasoning, the plot of *IP* versus *R*_inh_, both divided by the molar concentration of the antioxidants to take into account their different molecular weights, shows the presence of a general trend consisting of an inverse relation between *IP* and *R*_inh_ (see Figure 5), although the rather scattered data points suggest that other factors contribute to the *IP* vs. *R*_inh_ relationship.

It should also be pointed out that the phenomenon of chain propagation by the phenoxyl radicals of the antioxidants A^•^ is not observed at low temperatures when the autoxidation of SSO is accelerated by the decomposition of azoinitiators, probably because the high flux of ROO^•^ radicals ensures an efficient decay of A^•^ for all antioxidants, while the low temperature slows down reaction 9 [31]. On the other hand, the low *IP* of α-tocopherol compared to γ-tocopherol in soybean and sunflower oils at 100 °C has been in part attributed to reaction 9 [32]. Moreover, the chain propagation by the α-tocopheroxyl radicals is responsible for the tocopherol-mediated peroxidation in low-density lipoproteins [33].

**Scheme 5:**
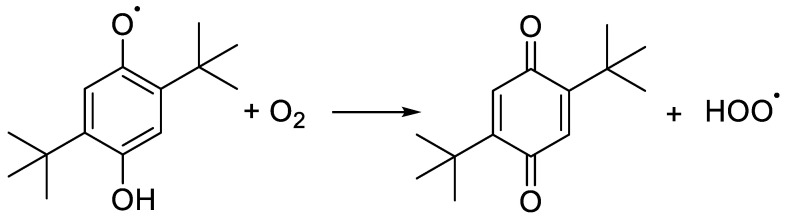
Chain propagation of dialkylated semiquinone radicals.

The low efficacy of the dialkylated hydroquinone **11** is instead to be ascribed to the tendency of the electron-rich semiquinones to react with O_2_ forming *para*-benzoquinones and HOO^•^, which propagates the oxidative chain (Scheme 5) [34].

## 5. Conclusions

Autoxidation experiments at 130 °C of stripped sunflower oil in the presence of various phenolic antioxidants, having monophenolic, hydroquinone, or catechol active moieties, showed that hydroquinones with one alkyl substituent display the best activity in terms of inhibition degree of O_2_ consumption and duration of the inhibited period. By using EPR spectroscopy, we could demonstrate that the stability of the radical of the antioxidants plays a counterintuitive role in determining the overall activity. Antioxidants like α-tocopherol, which form relatively persistent radicals, are not very effective in counteracting oil peroxidation, because their radicals can abstract an H-atom from polyunsaturated lipids. On the other hand, hydroquinones form very unstable semiquinone radicals that disappear quickly by H-atom donation to another radical, and thus are incapable of propagating the oxidative chain.

Although many efforts are still needed to fully clarify the mechanisms of the inhibition of lipid peroxidation, these results are important to develop novel antioxidant strategies for the protection of oils with a high polyunsaturated fatty acid content at high temperatures, and possibly of lipid-containing foods in general. Considering the interest in finding new sources of natural phenolic antioxidants, knowledge of the structural factors underlying their activity can drive the research toward new (poly)phenolic materials having hydroquinone functionality [35] with practical applications for food and biodiesel stabilization.

## Data Availability

Data is contained within the article.
